# Three-Dimensional Echocardiography Based on Automation and Machine Learning Principles and the Renaissance of Cardiac Morphometry

**DOI:** 10.3390/jcm11154357

**Published:** 2022-07-27

**Authors:** Andrea Barbieri, Mauro Pepi

**Affiliations:** 1 Division of Cardiology, Department of Diagnostics, Clinical and Public Health Medicine, Policlinico University Hospital of Modena, University of Modena and Reggio Emilia, 41124 Modena, Italy; 2 Centro Cardiologico Monzino IRCCS, 20138 Milan, Italy; mauro.pepi@cardiologicomonzino.it

Today, the core component of all transthoracic echocardiography reports is the quantification of cardiac chamber size and function using advanced echocardiography modalities such as three-dimensional echocardiography (3DE), in line with the rising demand for quantifications of cardiac chambers with high measurement accuracy and reproducibility. Competing against this need is the additional constraints on the echocardiography laboratory’s time and resources, such as the rise in the average study’s image acquisition rate and the requirement to include new measures. There is an understandable tendency to forego traditional image capturing due to concerns about missing corroborating information, as highlighted in a recent EACVI Scientific Initiatives Committee survey. Surprisingly, several centers that do not regularly employ 3DE in the morpho-functional evaluation of cardiac patients reported that they did not think the 3DE technique added any extra value [1].

Against this background, the real question is how automated morphology and function assessment with the assistance of artificial intelligence technology will change our practice, considering that in many cases, cardiac morphometry is still estimated qualitatively by eye or quantitatively using conventional bi-dimensional echocardiography (2DE).

It is always helpful to remind ourselves that the visual interpretation of cardiac chamber volumes and function has well-known shortcomings, independent of the operators’ experience [2]. As a result, referring physicians increasingly expect quantitative assessments. However, reading several echocardiograms is time-consuming and laborious because of the vast volume of information. Notably, manual measurements are known to differ significantly amongst readers. The poor acoustic window, the need for geometric modeling, and errors caused by foreshortened views, mistiming end-systole, or mistracing of the endocardial border represent notable limitations of conventional 2DE. Even minor variations affect downstream calculations of cardiac chambers [3].

Despite filtering out every known source of error, one of the critical factors of interobserver variability is the operator’s impression of the blood–tissue interface, with the existence of trabeculation being the primary source of disagreement, considering that trabeculae and papillary muscles accounted for as much as 23% of left ventricular (LV) volume [4]. Indeed, the LV volume assessment does change significantly depending on whether the operator draws the boundary by the blood tissue interface (i.e., at the tip of the trabeculation) or on the compact myocardium [5]. The latter value is more similar to the mean value determined by cardiac magnetic resonance imaging, which is presently regarded as the gold standard for quantifying heart chambers [6]. Delineation errors in these anatomic features can significantly impact LV and right ventricular (RV) volume and mass assessments, depending on how readily trabeculae and papillary muscles can be distinguished from myocardial tissue. Of note, differences as small as 1 mm in tracing the LV border produced 11% differences in volume measurements [4].

Individuals and institutions must standardize their LV border tracing practices to address this issue.

The use of machine learning techniques to automate echocardiograms overcomes many of these drawbacks and may revolutionize the workflow in echocardiography laboratories, promoting the use of 3DE measurements in busy laboratories. One of the automation’s major advantages is its excellent reproducibility, able to reduce the gap in reliability between expert and novice readers; given the same image, a completely automated software will always output the same endocardial border and volume. Other benefits of automation include reduced time and costs related to image acquisition, rapid analysis, and reporting [7]. Moreover, using an average of several observations to estimate chamber size or quantify velocities, gradients, or stroke volumes is helpful regardless of the underlying rhythm [8].

A meta-analysis shows that fully automated software outperforms semiautomated and manual software because individual interpreters’ positioning and editing endocardial contours introduce more substantial variability and bias [9]. However, 3DE-derived volumes are still underestimated in most patients because they cannot differentiate between compact myocardium and trabeculae, contributing to the persistent underestimation in values compared with cardiac magnetic resonance. Furthermore, the software still has difficulty accurately delineating the endocardial border of LV in some patients. For example, the shape and/or complex contraction patterns, such as multiple regional motion abnormality, are not in the training dataset [10].

Recently, a new generation of fully automated software based on an adaptive analytics algorithm has been developed for left-heart chamber quantification [11]. This novel technology appears to be feasible, fast, and reproducible [12,13,14,15]. The acquisition of LV foreshortened views is recognized by the current version of the software package, and several LV and left atrial (LA) function indices are automatically calculated from a single dataset in ~30″ [15]. Moreover, the user specifies where the final single endocardial border should be placed, allowing to bring the measurements obtained with 3DE ever closer to those obtained with cardiac magnetic resonance [12].

Simultaneously, incorporating 3DE into daily workflow necessitates the implementation of critical resources such as appropriate equipment (transducers, ultrasound machine, and software), patient selection, assimilation of 3DE protocols into current clinical routine, laboratory workflow adaptation, storage, and reporting. Once leadership and hospital administration have decided to acquire a 3DE system, the echo laboratory must establish an agreement on standardized 3DE methods. Our approach was to integrate 3DE within a 2DE protocol from the beginning if a 3DE probe was available and apply it routinely to all patients to evaluate left chambers and selectively for the right chambers. Our echo lab’s rate of acquiring a 3DE LV and LA volume is about 80%, including consecutive inpatient and outpatient studies [15,16]. Based on our and other [17] experiences, six major obstacles remain that prevent a wider spread of 3DE: (1) the underappreciation of the added clinical value of 3DE vs. conventional 2DE, (2) the finite number of 3DE probes available, (3) the need to organize a formal program of training and competency, (4) the presence of still unresolved technical issues of poor image quality, (5) the propriety algorithms to post-process and analyze 3DE datasets, and (6) the way to communicate the clinical significance of reporting changes from 2DE to 3DE, which remains to be defined.

It is crucial to understand that every cut-off number we use to determine whether to recommend valve surgery, device installation, etc. comes from controlled randomized studies that employed 2DE to calculate LV ejection fraction values. Nonetheless, the reference values for left heart chamber volumes assessed with 3DE fully automated software and conventional 2DE are quite different and cannot be utilized interchangeably [18]. As a result, echocardiography reports must be produced differently depending on the method employed and referring physicians must understand distinct cut-off values to distinguish between normal and enlarged volumes and function.

The creation of proprietary algorithms to post-process and analyze 3DE information poses an additional problem in multi-vendor echo labs, especially multi-site laboratories, where the staff must adapt to a different workflow.

In our experience, the most critical clinical challenge is represented by the substantial underestimation of LV volumes (and stroke volume), as determined by 2DE, compared to the values obtained with 3DE, especially in patients with dilated ventricles. When 3DE is performed during follow-up on patients whose earlier echo examination had been carried out with 2DE, our laboratory labels these data resulting from 3DE or 2DE as not directly comparable measurements.

However, perhaps the major limitation for the spread of 3DE is represented by the still too popular belief that artificial intelligence threatens our professionalism. The practice of echocardiography is seen as a combination of talent and science strongly dependent on the skills and experience of the operators. Conversely, we believe that the recent development of novel techniques to make an automated quantitative analysis of 3DE based on machine learning principles offers a “game-changer” opportunity to reduce the workload in our busy echo lab, improving the reproducibility and repeatability of the measurements [19].

Significantly, the high-performance, automated, adaptive analytics software offers the most accurate and reproducible quantitation of volumes, geometry, and function of the left heart and has the great potential to revolutionize our practice expanding potential clinical applications in ways that were inconceivable until recently. Indeed, we have at our disposal new algorithms for 3DE LV mass analysis [16]. Furthermore, these algorithms overcome many pitfalls of the conventional 2DE volumetric method, for example, for proper quantification of the mitral regurgitation, and the correct diagnosis of the low-flow state [20]. In addition, they eliminate the geometric assumptions regarding the LA geometry and the systematic foreshortening of the LA apical view [21,22].

The application of the advanced echocardiography modalities is equally indicated in the evaluation of the right chambers considering that the functional assessment of the RV on 2DE is primarily based on qualitative measures that are relatively easy to measure but subject to many limitations [23]. Recent improvements in 3DE semiautomated algorithms software analysis on board the echocardiographic scanners allow for fast and reproducible RV [24] and right atrial volumetric analysis [25]. It is easily predictable that, as data accumulate and user-friendly software packages become available, 3DE will gradually become common even in the morphometrics assessment of the RV [26] and RV–pulmonary arterial coupling [27].

However, embracing new technology such as 3DE necessitates a willingness to adapt based on the recognition that it adds value to our clinical practice and is simple to use, but too many echocardiographers are still unaware that with the new automated 3DE tools, it is possible to obtain a quantitative morphometric evaluation of all heart chambers on top of conventional 2DE in 38 ± 0.16 s, with a total acquisition time of 14.24 ± 3.32 min [28].

We will therefore have to be prepared for the inevitable conceptual revolution already underway in which physicians will be called to start their interpretation of transthoracic echocardiography with multiple automatically measured static/dynamic numerical indices alongside the images. While challenges remain, the transition from the 2DE to 3DE era is already real in 2022 and represents an exciting research field that is yet to be explored.

Beyond validation of new analysis software packages, the next inevitable step of 3DE research should be establishing reference values and outcome data for multiparametric data cut-offs in various disease states. In a related manner, setting normal reference values is necessary for 3DE to gain acceptance in clinical practice [29].

## References

[1] Marsan N.A., Michalski B., Cameli M., Podlesnikar T., Manka R., Sitges M., Dweck M.R., Haugaa K. (2020). EACVI survey on standardization of cardiac chambers quantification by transthoracic echocardiography. Eur. Heart J. Cardiovasc. Imaging.

[2] Cole G.D., Dhutia N.M., Shun-Shin M.J., Willson K., Harrison J., Raphael C.E., Zolgharni M., Mayet J., Francis D.P. (2015). Defining the real-world reproducibility of visual grading of left ventricular function and visual estimation of left ventricular ejection fraction: Impact of image quality, experience and accreditation. Int. J. Cardiovasc. Imaging.

[3] Yuan N., Jain I., Rattehalli N., He B., Pollick C., Liang D., Heidenreich P., Zou J., Cheng S., Ouyang D. (2021). Systematic Quantification of Sources of Variation in Ejection Fraction Calculation Using Deep Learning. JACC Cardiovasc. Imaging.

[4] Mor-Avi V., Jenkins C., Kühl H.P., Nesser H.-J., Marwick T., Franke A., Ebner C., Freed B.H., Steringer-Mascherbauer R., Pollard H. (2008). Real-time 3-dimensional echocardiographic quantification of left ventricular volumes: Multicenter study for validation with magnetic resonance imaging and investigation of sources of error. JACC Cardiovasc. Imaging.

[5] Chuang M.L., Gona P., Hautvast G.L., Salton C.J., Blease S.J., Yeon S.B., Breeuwer M., O’Donnell C.J., Manning W.J. (2012). Correlation of trabeculae and papillary muscles with clinical and cardiac characteristics and impact on CMR measures of LV anatomy and function. JACC Cardiovasc. Imaging.

[6] Landi A., Faletra F.F., Pavon A.G., Pedrazzini G., Valgimigli M. (2021). From secondary to tertiary mitral regurgitation: The paradigm shifts, but uncertainties remain. Eur. Heart J. Cardiovasc. Imaging.

[7] Nolan M.T., Thavendiranathan P. (2019). Automated Quantification in Echocardiography. JACC Cardiovasc. Imaging.

[8] Guta A.C., Badano L.P., Ochoa-Jimenez R.C., Genovese D., Previtero M., Civera S., Ruocco A., Bettella N., Parati G., Muraru D. (2019). Three-dimensional echocardiography to assess left ventricular geometry and function. Expert Rev. Cardiovasc..

[9] Kitano T., Nabeshima Y., Otsuji Y., Negishi K., Takeuchi M. (2019). Accuracy of Left Ventricular Volumes and Ejection Fraction Measurements by Contemporary Three-Dimensional Echocardiography with Semi- and Fully Automated Software: Systematic Review and Meta-Analysis of 1,881 Subjects. J. Am. Soc. Echocardiogr..

[10] Muraru D., Cecchetto A., Cucchini U., Zhou X., Lang R.M., Romeo G., Vannan M., Mihaila S., Miglioranza M.H., Iliceto S. (2018). Intervendor Consistency and Accuracy of Left Ventricular Volume Measurements Using Three-Dimensional Echocardiography. J. Am. Soc. Echocardiogr..

[11] Tsang W., Salgo I.S., Medvedofsky D., Takeuchi M., Prater D., Weinert L., Yamat M., Mor-Avi V., Patel A.R., Lang R.M. (2016). Transthoracic 3D Echocardiographic Left Heart Chamber Quantification Using an Automated Adaptive Analytics Algorithm. JACC Cardiovasc. Imaging.

[12] Tamborini G., Piazzese C., Lang R.M., Muratori M., Chiorino E., Mapelli M., Fusini L., Ali S.G., Gripari P., Pontone G. (2017). Feasibility and Accuracy of Automated Software for Transthoracic Three-Dimensional Left Ventricular Volume and Function Analysis: Comparisons with Two-Dimensional Echocardiography, Three-Dimensional Transthoracic Manual Method, and Cardiac Magnetic Resonance Imaging. J. Am. Soc. Echocardiogr..

[13] Medvedofsky D., Mor-Avi V., Amzulescu M., Fernández-Golfin C., Hinojar R., Monaghan M.J., Otani K., Reiken J., Takeuchi M., Tsang W. (2018). Three-dimensional echocardiographic quantification of the left-heart chambers using an automated adaptive analytics algorithm: Multicentre validation study. Eur. Heart J. Cardiovasc. Imaging.

[14] Narang A., Mor-Avi V., Prado A., Volpato V., Prater D., Tamborini G., Fusini L., Pepi M., Goyal N., Addetia K. (2019). Machine learning based automated dynamic quantification of left heart chamber volumes. Eur. Heart J. Cardiovasc. Imaging.

[15] Italiano G., Tamborini G., Fusini L., Mantegazza V., Doldi M., Celeste F., Gripari P., Muratori M., Lang R.M., Pepi M. (2021). Feasibility and Accuracy of the Automated Software for Dynamic Quantification of Left Ventricular and Atrial Volumes and Function in a Large Unselected Population. J. Clin. Med..

[16] Barbieri A., Bursi F., Camaioni G., Maisano A., Imberti J., Albini A., De Mitri G., Mantovani F., Boriani G. (2021). Echocardiographic Left Ventricular Mass Assessment: Correlation between 2D-Derived Linear Dimensions and 3-Dimensional Automated, Machine Learning-Based Methods in Unselected Patients. J. Clin. Med..

[17] Hur D.J., Sugeng L. (2020). Integration of three-dimensional echocardiography into the modern-day echo laboratory. Echocardiography.

[18] Addetia K., Miyoshi T., Amuthan V., Citro R., Daimon M., Fajardo P.G., Kasliwal R.R., Kirkpatrick J.N., Monaghan M.J., Muraru D. (2022). Normal Values of Left Ventricular Size and Function on Three-Dimensional Echocardiography: Results of the World Alliance Societies of Echocardiography Study. J. Am. Soc. Echocardiogr..

[19] Donal E., Muraru D., Badano L. (2021). Artificial intelligence and the promise of uplifting echocardiography. Heart.

[20] Patel H.N., Miyoshi T., Addetia K., Henry M.P., Citro R., Daimon M., Fajardo P.G., Kasliwal R.R., Kirkpatrick J.N., Monaghan M.J. (2021). Normal Values of Cardiac Output and Stroke Volume According to Measurement Technique, Age, Sex, and Ethnicity: Results of the World Alliance of Societies of Echocardiography Study. J. Am. Soc. Echocardiogr..

[21] Badano L.P., Miglioranza M.H., Mihăilă S., Peluso D., Xhaxho J., Marra M.P., Cucchini U., Soriani N., Iliceto S., Muraru D. (2016). Left Atrial Volumes and Function by Three-Dimensional Echocardiography: Reference Values, Accuracy, Reproducibility, and Comparison With Two-Dimensional Echocardiographic Measurements. Circ. Cardiovasc. Imaging.

[22] Thomas L., Muraru D., Popescu B.A., Sitges M., Rosca M., Pedrizzetti G., Henein M.Y., Donal E., Badano L.P. (2020). Evaluation of Left Atrial Size and Function: Relevance for Clinical Practice. J. Am. Soc. Echocardiogr..

[23] Longobardo L., Suma V., Jain R., Carerj S., Zito C., Zwicke D.L., Khandheria B.K. (2017). Role of Two-Dimensional Speckle-Tracking Echocardiography Strain in the Assessment of Right Ventricular Systolic Function and Comparison with Conventional Parameters. J. Am. Soc. Echocardiogr..

[24] Surkova E., Cosyns B., Gerber B., Gimelli A., La Gerche A., Ajmone Marsan N. (2022). The dysfunctional right ventricle: The importance of multi-modality imaging. Eur. Heart J. Cardiovasc. Imaging.

[25] Lang R.M., Cameli M., E Sade L., Faletra F.F., Fortuni F., Rossi A., Soulat-Dufour L. (2022). Imaging assessment of the right atrium: Anatomy and function. Eur. Heart J. Cardiovasc. Imaging.

[26] Muraru D., Badano L.P. (2022). Shedding new light on the fascinating right heart. Eur. Heart J. Cardiovasc. Imaging.

[27] Yuchi Y., Suzuki R., Higuchi R., Saito T., Teshima T., Matsumoto H., Koyama H. (2022). Utility of Real-Time Three-Dimensional Echocardiography for the Assessment of Right Ventricular Morphology and Function in Large Animal Models. J. Clin. Med..

[28] Volpato V., Ciampi P., Johnson R., Hipke K., Tomaselli M., Oliverio G., Muraru D., Badano L.P., Lang R.M. (2022). Feasibility and time-analysis of 3D and myocardial deformation versus conventional 2D echocardiography to assess cardiac chambers. J. Am. Soc. Echocardiogr..

[29] Bernard A., Addetia K., Dulgheru R., Caballero L., Sugimoto T., Akhaladze N., Athanassopoulos G.D., Barone D., Baroni M., Cardim N. (2017). 3D echocardiographic reference ranges for normal left ventricular volumes and strain: Results from the EACVI NORRE study. Eur. Heart J. Cardiovasc. Imaging.

